# Examining the Determinants of Patient Perception of Physician Review Helpfulness across Different Disease Severities: A Machine Learning Approach

**DOI:** 10.1155/2022/8623586

**Published:** 2022-02-26

**Authors:** Adnan Muhammad Shah, Wazir Muhammad, KangYoon Lee

**Affiliations:** ^1^Department of Computing Engineering, Gachon University, Seoul 13120, Republic of Korea; ^2^Department of Physics, Charles E. Schmidt College of Science, Florida Atlantic University, Boca Raton, FL 33431-0991, USA

## Abstract

(1) *Background*. Patients are increasingly using physician online reviews (PORs) to learn about the quality of care. Patients benefit from the use of PORs and physicians need to be aware of how this evaluation affects their treatment decisions. The current work aims to investigate the influence of critical quantitative and qualitative factors on physician review helpfulness (RH). (2) *Methods*. The data including 45,300 PORs across multiple disease types were scraped from *Healthgrades.com*. Grounded on the signaling theory, machine learning-based mixed methods approaches (i.e., text mining and econometric analyses) were performed to test study hypotheses and address the research questions. Machine learning algorithms were used to classify the data set with review- and service-related features through a confusion matrix. (3) *Results*. Regarding review-related signals, RH is primarily influenced by review readability, wordiness, and specific emotions (positive and negative). With regard to service-related signals, the results imply that service quality and popularity are critical to RH. Moreover, review wordiness, service quality, and popularity are better predictors for perceived RH for serious diseases than they are for mild diseases. (4) *Conclusions*. The findings of the empirical investigation suggest that platform designers should design a recommendation system that reduces search time and cognitive processing costs in order to assist patients in making their treatment decisions. This study also discloses the point that reviews and service-related signals influence physician RH. Using the machine learning-based sentic computing framework, the findings advance our understanding of the important role of discrete emotions in determining perceived RH. Moreover, the research also contributes by comparing the effects of different signals on perceived RH across different disease types.

## 1. Introduction

Understanding patient preferences of service quality is vital for the healthcare industry and healthcare providers to develop optimal strategies to improve patients' quality of care [1]. With the growing popularity of physicians rating websites (PRWs), better information can be obtained regarding factors influencing patients' choices of selecting the right doctor [2]. Unlike traditional surveys used to collect information on patients' preferences and treatment experiences, physician online reviews (PORs) offer a rich source of knowledge without the interventions by researchers or healthcare organizations [3]. Recent studies have shown that PORs are specific type of word of mouth that plays a crucial part in the patients' decision-making [4]. PORs are a significant source of knowledge for many patients who are looking for a good doctor [5]. They see these PRWs as an important source for finding the best doctor [6, 7]. These PORs offer authentic information for patients' wellbeing but likewise contribute to an evolving relationship between doctors and their patients [8, 9].

Using feature engineering to identify helpful reviews, users could be able to reduce the search cost. Although PORs alleviate the overall choice burden on users, they also trigger many issues, such as presenting misguided or inappropriate information [10]. Hence, it is critical to explore review helpfulness (RH) by identifying the characteristics of highly helpful reviews. In the previous research, the review assessment was mainly focused on quantitative characteristics of a review (e.g., rating, valence, and sentiment polarity) [11, 12]. Shah et al. [13] indicated that quality of service in terms of dispersion of online reviews significantly influences RH. Researchers also indicated that reviews for popular services get more helpful votes [10, 14]. Moving away from quantitative measures, more recent research focused on qualitative measures (e.g., readability, word count, and emotions) to evaluate RH [11, 12, 15, 16]. In considering the multiple review types and related key issues, however, helpfulness is quite a nuanced term, as quantitative measures of reviews are equally useful, whereas others might consider qualitative characteristics as more helpful [15]. Fang et al. [17] indicated that text readability significantly influences perceived RH. Mauro et al. [18] revealed that review wordiness is a meaningful predictor of RH. The study of Malik and Hussain [19] indicated that discrete emotions are the most dominant emotions with greater influence on perceived RH. Ren and Hong [20] found a significant relationship between negative emotions and RH. This reveals that various aspects of information and behaviors are helpful in the desirable purchase decision-making process [21–23].

For quite a while, the research topic of RH has drawn academic interest in the search and experience goods context [19, 20, 24]. The idea of RH has also gained researchers' attention in the healthcare domain, which is categorized as credence goods. In comparison to other goods, credence goods are distinct since the quality of credence goods cannot be determined even after the utility has been consumed [25]. Evaluating the effectiveness of PORs in comparison to product and service reviews is challenging, as the provider defines the utility impact of the goods, creating an asymmetric information state. The findings from previous studies revealed that, for patients, the helpfulness of PORs plays a pivotal part in their treatment decision-making process and for a physician to improve the quality of care [13, 22, 23, 26]. However, RH was not thoroughly investigated as a function of quantitative and qualitative measures concurrently in credence goods context (healthcare). This research was therefore conceived to expand earlier studies on the RH by exploring not only the quantitative factors (valence and volume), but qualitative characteristics of reviews as well such as readability features, wordiness features, and discrete emotions. Hence, this study classifies different review attributes (concept-level prospective) and service characteristics and implicitly assesses patients' distinct emotions by employing sentic computing model proposed by Cambria et al. [27] to compute the physician RH.

In addition, patients' perception of physician service quality varies across two main disease types: serious diseases (high disease severity) and mild diseases (low disease severity). Disease severity determines how severe the effects of a type of illness are [28–30]. Because the disease severity is normally associated with unspecified factors such as unexpected mortality, increased treatment costs, and prolonged hospital stays, patients with more severe disease tend to be more anxious about the quality of treatment they receive from their physicians than those with less severe disease [8]. Our approach shows that the review- and service-related signals are highly associated with the helpfulness count of PORs that affect decision-making of patients who suffer from different disease types (high-illness severity vs. low-illness severity). Using the plethora of information in terms of PORs provided by *Healthgrades.com* and users of the online healthcare services they choose for their cure (PORs), we applied a text mining and econometric approach to determine the signaling mechanisms that affect patients' treatment choice of different physicians. Additionally, secondary data analysis and machine learning classification approaches were used to construct more accurate models for predicting physician RH. As a result of these considerations, this study will attempt to answer the subsequent research questions (RQs):

 
**RQ1.** What effect do the various review- and service-related signals have on the physician RH?
 
**RQ2.** How do specific emotions (joy, sadness, surprise, trust, anger, anticipation, disgust, and fear) associated with review-related signals influence physician RH implicitly?
 
**RQ3.** What role does disease type have in the relationships between review- and service-related signals and physician RH?

This work contributes to the digital health literature by differentiating between the two kinds of signals (review- and service-related signals) and exploring their effects on online physician RH. Drawing on signaling theory, the research contributes to the conceptualization and interpretation of the RH features from both quantitative and qualitative perspectives. The dataset, including 45,300 PORs from *Healthgrades*, examined ten hypotheses. The proposed model was successfully validated, and critical components that would make an opinion relevant to readers were discovered. The study findings have added to related literature by offering more comprehension of the structural characteristics (quantitative and qualitative) of reviews and their effect on RH [18, 19, 31]. The findings indicate that both the review- and service-related signals significantly and positively influence perceived RH. Second, following an examination of the differential impacts on physician RH, we examine the effect of distinct emotions (sentiments) on RH. Despite the fact that extant research has been conducted on the role of emotions expressed in PORs [22, 32, 33], the research on the domain knowledge-based specialized discrete emotion analysis of online textual reviews has been neglected. This study examines the impact of two-sided discrete emotions embedded in PORs on physician RH. The results show that two-sided reviews (positive and negative discrete emotions) significantly influence the perceived RH. Third, this study utilized the idea of environmental uncertainty, which has been referred to as disease type in the virtual healthcare market setting in prior strategic studies [22, 26, 28, 33, 34]. This study extends the scope of prior research and advances signaling theory by examining how the effects of review- and service-related signals on physician's RH differ according to the type of disease being treated. The findings revealed a significant positive moderating effect of disease type in the relationships between review wordiness, service quality, service popularity, and perceived RH. We focus on multimethod analysis, including implicit and domain knowledge-based specialized sentic computing emotion analysis and econometric approach to predict physician RH. The proposed multimethod model shows an excellent performance with a classification accuracy of 91.12%.

## 2. Theoretical Background

RH refers to how many times the review has been voted as useful by other reviewers in order to guide purchase decisions [35]. The confounding variation in reviews posted for one commodity makes it challenging for customers to evaluate helpful entities. The sheer number of competitive goods and overwhelmed data make it difficult for consumers' online decision-making. PRWs like RateMDs and Healthgrades were the pioneer platforms of helpful voting to mitigate the problem.

Particularly, this feature explicitly leverages crowdsourcing to determine the helpfulness of reviews. A question follows each review, “*Was this review helpful?*” Consumers who have read reviews might vote by clicking the option: Yes or No. Reviews receiving optimistic or crucial helpfulness votes are followed by notes, for instance, “*This review was useful to 6 out of the 9 people who read it.*” The most helpful satisfactory and negative reviews for a single commodity finally top the list of reviews on rating sites. Online rating sites rank customer feedback based on helpfulness score, which minimizes the customers' time to find valuable information [36]. Helpful voting, together with customer feedback, provides a broad range of information in which researchers can look at factors that could influence consumers' purchasing decisions in an e-shopping setting [37]. Consequently, improving the RH is positively linked to product sales, mainly if they are favorable [14]. The helpfulness voting feature has been of great scientific interest over time. For example, the main components of RH such as review valence [12, 38], volume [14], depth of a review [15, 16, 39], linguistic features [23, 40], readability [11], and emotions [41, 42] have been used to predict RH.

Many PRWs have set up peer-review systems that let people make healthcare decisions based on whether they found a review helpful [13]. For example, *Healthgrades.com* offers a service that presents the top two most-rated reviews submitted by online health consumers to assist other customers in evaluating the quality of physician care. These helpful votes are used as a proxy for review diagnosticity, allowing for the separation of helpful and unhelpful reviews [38]. To put it another way, the helpful information contained in a review may help the health consumers to evaluate the attributes of the physician service quality. This means that Internet information sources with more useful reviews can help patients feel more confident about their consultation intentions [43]. Consistent with this perspective in healthcare, the patients' behavior and interests also shift across different disease type. For example, a patient with a high-risk disease receives different levels of care than a patient with low-risk disease. Serious disease patients could be more vulnerable to the healthcare quality than patients suffering from common diseases. Previous research has shown that individual health condition has a major impact on their decision to visit a healthcare professional [8, 28, 44].

The literature on RH focuses on the economics of knowledge and how it changes the purchaser buying decision-making process in order to lessen purchase uncertainty associated with the product [38]. The claim made in prior investigations is complemented by signaling theory, which provides a theoretical framework for explaining the differential influence of signals in PORs. In this investigation, we used signaling theory to describe the relationship between signals enclosed in PORs and RH. According to signaling theory, signals contribute to diminishing the information asymmetry between two transaction contributors. Spence [45] demonstrated that information asymmetry exists between various exchange groups when information is exchanged. Signals are important in an online environment because they help minimize the information gap as spatial and temporal gaps make information asymmetry worse between distinct partners [46]. Signal receivers are critical components of the signaling cycle. As a result, the *sender* communicates with the *receiver* via information (*signals*), which the receiver perceives as useful information [47]. The substantial impact of Internet information on stakeholders' decision-making demonstrates that the more the knowledge an individual possesses, the more improved the decision s/he will make [48].

Signaling theory contributes to the reduction of information asymmetry between physicians and patients. People with less knowledge about the credibility of a healthcare provider tend to find information from people who know a lot about it. Thus, peer perspectives can help evaluate information quality and minimize information asymmetry [28]. Previous researchers have used the signaling theory to explore numerous signals in healthcare [25, 28]. While earlier research has largely focused on the sender's insight while neglecting the receiver's opinion, the bond between various signal-related elements could substantially impact the receiver's experience as RH. Signaling theory [28] states that the receiver (*patient*) requires supplementary information (*signals*) regarding the quality of healthcare to reduce information asymmetry prior to contacting their provider.

Signaling theory posits that uncertainty in the environment could have a big impact on how people process signals. According to signaling theory, the impact of various signal transmissions on a patient's choice differs between various settings [25]. The signaling environment is crucial in determining which signal to employ, and the strength of the signal is controlled by the signaling ecosystem in which it operates [47]. The intended recipients of the signal are users who are interested in learning more about healthcare services like RH. Keeping in view the user-generated content, the availability of PORs may have an impact on the examination of which elements influence RH under varied illness circumstances (disease severity).

Although the main objective of the above discussion was to improve the framework of the online review to promote more helpfulness votes, little has been done to explore how the interplay of review- and service-related factors is related to POR's helpfulness. No consistent conclusions have been drawn yet regarding the important factors influencing POR's helpfulness. In addition, the role of the disease type in the RH is yet to be widely explored. Our work fills these knowledge gaps.

## 3. Hypotheses Development

**Figure 1:**
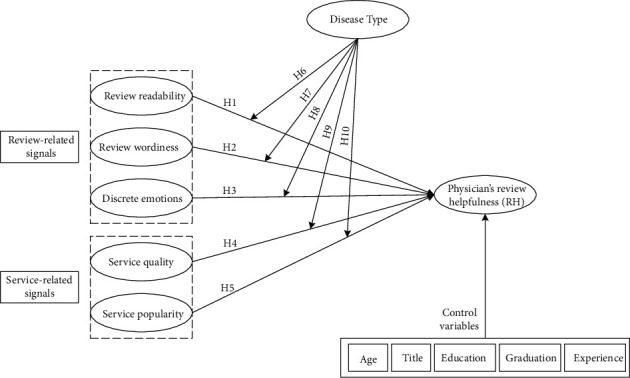
Research model.

This work proposes research hypotheses to examine the influence of various review- and service-related signals on physician RH. The former considers three features: readability, wordiness, and discrete emotions, while the latter takes into account the service quality and popularity. Lastly, this work examines the extent to which patients' assessments of a physician RH vary among distinct disease types.  Figure 1 sets out the research model.

The readability of a review implies how easily a reader may comprehend a piece of writing. Online reviews must be comprehensible when used as an input variable in order to make buying decisions [19]. Extant research indicated that the level of readability is how well an individual follows the product information [11]. Readability has been identified as a significant component in customers' perception of online information on virtual networks. A review that is sufficiently readable is deemed more beneficial to consumers than one that is excessively lengthy and contains several typographical errors, making it difficult to read [12, 49]. Following readable reviews could help patients save a search and cognitive costs by finding the right information easier [23]. Hence, we hypothesize that the more understandable the text is on the health rating platform, the more useful the review is.

**Hypothesis 1 .**

**(H1)**. *The higher readability of PORs is positively related to higher RH votes.*

The idea of a review wordiness is usually thought of as the amount of information in a review that is detailed or long [23, 50]. It has been demonstrated by researchers that decision-makers capacity to comprehend information is hampered when the amount of data is either excessively high or excessively low. Insufficient information has a negative impact on buyer decisions [39]. Previous studies have also revealed that extreme or knowledge overload can have an adverse effect on the RH in some people [15]. The length of the review is regarded to be an important predictor of RH [16, 38]. According to previous research, the wordiness of a review is considered helpful and acts as directly proportional to the amount of knowledge generated by a review. However, in the event of excessive repetition of the concept, misconceptions, and needless details, wordiness may lead to a poor assessment of helpfulness [15]. Hence, following longer reviews when it provides extensive information may diminish patients' search costs due to enhanced information diagnosticity [26]. Therefore, we have the following hypothesis.

**Hypothesis 2 .**

**(H2)**. *Review wordiness is positively related to higher RH votes.*

Emotions stated in PORs are relevant because they influence patients' clinical decisions [22, 28]. Emotions have been described as an appraisal of a shift in the feelings of a person [51]. Reviews that include both positive and negative sentiments about the appraisal of products or services are the best possible sources of information [51–53]. Researchers have claimed mixed findings of the emotions embedded in a review in positive-negative continuum with some researchers finding positive emotions are more useful [19, 22, 42], while other groups of scholars indicated negative emotions as more diagnostic and helpful [20, 54]. A review is considered as more significant because it gives clear signals about whether or not a service should be taken into account. Consequently, the specific emotion signals (i.e., joy, sadness, surprise, trust, anger, anticipation, disgust, and fear) have an effect on how health consumers perceive the RH [22]. Hence, we hypothesize the following.

**Hypothesis 3 .**

**(H3)**. *Discrete emotions embedded in PORs are positively related to higher RH votes.*

Using service characteristics, capabilities, and features, consumers analyze and form opinions regarding the real level of service they receive [55]. Service quality indicates how users think about the dominance or weakness of the services they use [10]. Patients' perceptions of service quality are shaped by information regarding the quality of the service they get from their peers [56]. For example, patients prefer to consult a physician with a higher star rating for the quality of care [8]. Service rating has been shown to have a positive correlation with RH [37, 41]. Keeping in view the scope of study, physicians' ratings show whether or not people have an excellent or negative perception of their doctors [33]. People are more likely to be drawn to high-quality services, and they are also more inclined to give positive feedback about their experiences [57]. Hence, we hypothesize the following.

**Hypothesis 4 .**

**(H4)**. *Physicians with exceptional service quality are positively related to higher RH votes.*

The popularity of service can be judged by the number of individuals who have talked about it and/or expressed interest in purchasing it [14]. In online health rating platforms, patients considered the amount of PORs to reflect the market or the reputation of a service based on how many people used it [25]. Patients' perceptions of the level of popularity on PRWs may support them in evaluating the quality of their treatment and predicting service delivery [56]. Moreover, a high volume of PORs enhances the likelihood of obtaining correct information that can assist patients in assessing the quality of healthcare services [58]. Researchers discussed that the more the information a patient has, the more likely s/he will make a better decision [59]. A well-known service entices additional users to read and vote on customer reviews. This means that patients may be more confident in assessing the quality and outcome of treatment if many individuals have already reviewed it. Hence, we hypothesize the following.

**Hypothesis 5 .**

**(H5)**. *Service popularity is positively related to higher RH votes.*

Information asymmetry theory states that the efficiency of signals transmission is dependent on the degree of environmental uncertainty. According to the findings of Siering et al. [47], signals have minimal effect on RH when the information environment uncertainty is low. On the other hand, relationships thrive in high uncertainty about information. Given that transmitting signals might help minimize uncertainty, it is evident that the usefulness of the signal is proportional to the degree of uncertainty.

It has also been assumed that the disease type as a feature moderates the association between readability and RH. In the services sector, a more readable review can be assessed more simply than a review with spelling mistakes and ambiguous words [17, 50]. Similarly, patients who suffer from serious diseases expect that information embedded in a review should be more readable than those who suffer from mild diseases. As follows, we posit that readable information about healthcare quality provided in PORs is more helpful for health consumers when they assess severe illnesses.

By adding confidence in the consumers' decision-making, longer reviews may be perceived as more helpful in the purchase process [38]. The detailed information provides further explanation about service and the context where the service was used. Wordiness has varying effects on the RH across different environments [10]. The added content in PORs is more likely to deliver crucial evidence about how the service is consumed and how it relates to alternatives [15]. Therefore, we assumed that patients need more detailed information about the quality of service for serious diseases than those who suffer from moderate illnesses.

This study also takes into account the disease type as a moderator of the relationship between individual emotions and RH. Patients with diverse illness conditions may require varying degrees of healthcare quality [8]. Those with a severe disease (high disease severity) may require a higher standard of care than patients with mild disease (low disease severity) [30]. As a result, we presume that specific emotions involved in PORs will be considered to provide comprehensive information, including service details for serious diseases compared to mild diseases [13, 23]. In this vein, a review that includes both good and negative emotions is likely to be more beneficial for serious illness, as these PORs are less distressing to readers who do not agree with the stated opinion. Keeping in view the earlier discussion, we hypothesize the following.

**Hypothesis 6 .**

**H6**. The moderating role of disease type in the relationship between the readability of a review and RH is stronger for serious diseases than it is for mild diseases.

**Hypothesis 7 .**

**H7**. The moderating role of disease type in the relationship between depth of a review and RH is stronger for serious diseases than it is for mild diseases.

**Hypothesis 8 .**

**H8**. The moderating role of disease type in the relationship between sentiment strength of a review and RH is stronger for serious diseases than it is for mild diseases.

Moving further, we hypothesize that if the disease severity is low, the influence of popularity and quality signals on physician RH will be minimal, owing to the fact that they do not properly minimize uncertainty. In comparison, physician RH will be strongly affected by both these signals if the disease severity is high since it greatly decreases the related uncertainty. Furthermore, patients with serious diseases are more likely to seek medical help from a doctor who offers high-quality and well-known services. Patients who have had a positive experience with a popular health service that is high-quality are more inclined to recommend the service to others and write reviews about it.

As a result of enhanced service awareness among health consumers, the likelihood of receiving high-quality service and helpful reviews increases because high-risk diseases necessitate a greater degree of service than low-risk diseases; high-risk diseases are connected with popular and high-quality services. Consequently, evaluating the characteristics of popular and high-quality services requires more effort than evaluating the traits of less popular and low-quality services. [60]. Hence, we hypothesize the following.

**Hypothesis 9 .**

**(H9)**. The moderating role of disease type in the relationship between service quality and RH is stronger for serious diseases than it is for mild diseases.

**Hypothesis 10 .**

**(H10)**. The moderating role of disease type in the relationship between service popularity and RH is stronger for serious diseases than it is for mild diseases.

## 4. Research Methodology

### 4.1. Research Context and Data Collection

**Figure 2:**
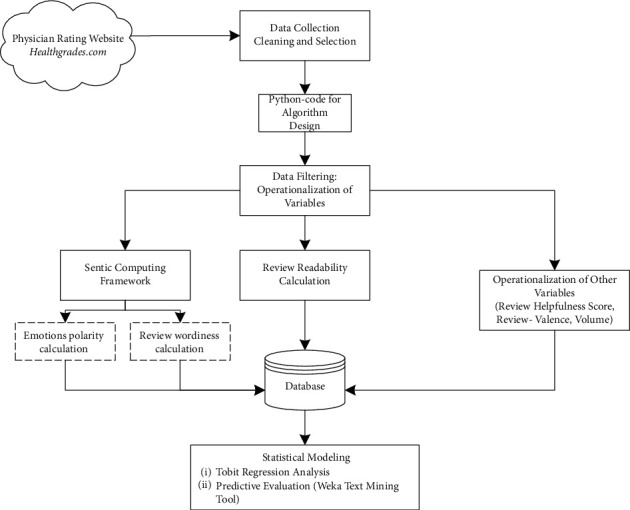
Research methodology followed.

The data was collected from an online health rating platform (*Healthgrades*) from March 15–21, 2019. Data preprocessing was performed in the form of filtering physician description, review posting date, online reviews, quantitative ratings, and helpfulness count. Online reviews are given further consideration to find out the readability score (six readability tests) and review wordiness using number of concepts in each review in the dataset from multiword expressions, such as “hospital corridor,” “operation theatre equipment,” or “physician appointment,” matched from SenticNet3 [27]. A hybrid sentic computing framework based on the text mining methodology was used to analyze the number of concepts from SenticNet3 linked to each specific emotion. In order to assess the overall performance of the proposed model in predicting RH, a number of regression analyses and text classifications were performed on data that had been filtered and cleaned up. The proposed methodology is shown in Figure 2.

A Web crawler was developed and programmed in Python 3.6 to retrieve the physician web pages shown as search results for each provider. The current study chose 10 different types of online reviews on the basis of disease mortality rate taken from the U.S. health static book 2017 [61] and 4 metropolitan states (California, New York, Texas, and Florida). According to data from the State Medical Boards, these states constitute the largest number of physicians with active board licenses. After omitting 236 reviews because of no helpful votes, 45,300 reviews were used for further analysis in total. The following information was gathered and included in the analysis, such as doctor specialty, title, education, experience, graduation year, review date, overall rating, number of ratings, patients' comments, and user responses (helpfulness or usefulness votes).

### 4.2. Variables Measurements and Statistical Modeling

#### 4.2.1. Review Helpfulness

A cumulative helpfulness vote is calculated on PRWs, and it is derived from the votes of other reviewers who rate the helpfulness level assigned to each individual review. When a POR gets more *helpfulness* votes, the review's *helpfulness* value rises as a result. *Review helpfulness* variable is assumed to be continuous and assessed as the ratio of helpful/useful votes to total votes. Nonvoted reviews were culled from our database in order to diminish the noise.

#### 4.2.2. Review Readability

Researchers revealed that RH could be influenced by the readability of online reviews [19, 49]. Ghose and Ipeirotis [49] revealed that the degree to which reviews contain subjectivity, knowledge, readability, and linguistic accuracy has an effect on their perceived usefulness. Six types of *readability* methods were explored for each review in order to assess its readability (refer to Table 1).

#### 4.2.3. Review Wordiness

The amount of concepts in a review is used to determine review wordiness [15]. Earlier research has established a substantial correlation between the review depth and RH [38, 62]. *Wordiness* is calculated using the sentic computing framework and SenticNet3 to measure the number of concepts in a review from multiword expressions. SenticNet 3 has previously been used to determine review wordiness as multiword expressions that make online content viral [15, 28].

#### 4.2.4. Service Quality and Service Popularity

Based on previous research [40], a collection of service-associated attributes is included, for instance, (1) *service quality* (i.e., service review valence) [63] and (2) *service popularity* (i.e., review volume), [14].

#### 4.2.5. Disease Type

Following [28], in disease type as a dummy variable, serious diseases as high-risk diseases are labeled as 0, while mild diseases as low-risk diseases are termed 1.

#### 4.2.6. Control Variables

We incorporate control variables to adjust for review- and physician-specific effects. As a control variable, the Review *Age* is provided to represent the distinctive qualities of a review [18]. The age of a review is how long it has been since it was written on online rating platform [39, 64]. Physician *title*, *education*, *graduation year*, and *experience* are the attributes displayed at the physician level. The *title* dummy variable measures the physician's professional title in the healthcare facility where s/he works [8]. We measured the *education* as a dummy variable using the medical school rank from where the physician graduated and *graduation year* as a categorical variable reflecting the number of years since s/he graduated [28]. Previous research has shown that physicians who graduated from prestigious medical schools are more likely to be rated highly. In comparison, the rating probability is lower for those younger physicians who graduated recently [28]. Finally, *experience* is defined as the period of time (in years) a physician has been in practice. In the past, more experienced physicians have been seen to receive higher ratings [8]. The description of variables and their measurements are listed in Table 1.

### 4.3. Sentic Computing Framework for Review Wordiness and Specific Emotions

Sentiment mining is a complex process that requires a thorough understanding of the goals of the study. Sentic computing has been applied to a variety of cognitively motivated tasks, including the classification of certain emotions (positive or negative) in natural language text [65]. Integrating knowledge-based methodologies and statistical methods, the hybrid approach to sentic computing and sentiment analysis is able to recognize emotions and calculate sentiments from the text [28]. The concept mining and emotion classification procedures used in this investigation are depicted in Figure 1.

We undertake preliminary textual data preprocessing, which includes the following: (i) Stop words have been removed (i.e., the, an, and a, an, etc.). (ii) Use the WordNetLemmatizer function and the WordNet Python natural language toolkit to convert a word to its base structure. (iii) Remove any unnecessary letters (i.e., thanks aloooooot). (iv) Question words should be filtered (i.e., which, whose, where, etc.) and any unique characters are excluded (&, #, $, etc.). (v) Finally, the entire text document is transformed to lower case.

We fragmented the review text into clauses first. Each verb and its corresponding noun phrase are deemed to extract one or more concepts. To make sentences more organized, the input text is chunked using Stanford Chunker [66]. Next, a semantic parser first breaks sentences into clauses and then employs a tree structure to divide clauses into noun and verb chunks [67]. Moreover, a two steps' procedure is followed for clause normalization: First, the Stanford lemmatization algorithm combined with WordNetLemmatizer function from WordNet NLTK is employed to normalize the *verb* chunks and to identify multiword expressions. Once the noun phrases have been converted into bigrams, they are processed using part-of-speech (POS) (https://nlp.stanford.edu/software/tagger.html) patterns to extract concepts as previously performed by Cambria and Hussain [68]. In addition, the event concept is captured by a parse graph; matches between the object concept and normalized verb chunks are explored in SenticNet3 [69]. Concepts are converted into a vector space model (VSM), where each concept is characterized by a point in a one-dimensional vector space corresponding to a vocabulary phrase.

The affective knowledge is represented using a multidimensional VSM. Concept Net and WordNetAffect have been used to create an Affective Space, a multidimensional vector space that illustrates lexical representations of affective knowledge. Affective space portrays the semantic and affective connections that exist between two concepts and allows for quick and effective analogical reasoning between them [68]. Equation (1) characterizes each document *d*, *C*_*i*_ denotes a concept in *d*, and *f*_*i*_ denotes the concept frequency in *d*.

(1) $$\begin{array}{c} d=\left\{\left(C_{1},f_{1}\right),\left(C_{2},f_{2}\right)\ldots ..\left(C_{i},f_{i}\right)\right\}. \end{array}$$

The cosine similarity between a concept's (*C*_*i*_) vector space representation (${\overset{⟶}{f}}_{i}$) and the vector space representation of the positive (${\overset{⟶}{f}}^{+}$) and negative context terms (${\overset{⟶}{f}}^{-}$) was obtained earlier, whereas, *n* denotes the total number of concepts in a document.

(2) $$\begin{array}{c} \text{Cosine\_sim}\left(C_{xy_{i}^{+}}\right)=\frac{{\overset{⟶}{f}}_{i}.{\overset{⟶}{f}}_{i}^{+}}{\left|f_{i}\right|.\left|f^{+}\right|}=\frac{\sum_{i=1}^{n}f_{xi}\times f_{yi}}{\sqrt{\sum_{i=1}^{n}f_{xi}^{2}}\sqrt{\sum_{i=1}^{n}f_{yi}^{2}}}\text{;}\\ \text{Cosine\_sim}\left(C_{xy_{i}^{-}}\right)=\frac{{\overset{⟶}{f}}_{i}.{\overset{⟶}{f}}_{i}^{-}}{\left|f_{i}\right|.\left|f^{-}\right|}=\frac{\sum_{i=1}^{n}f_{xi}\times f_{yi}}{\sqrt{\sum_{i=1}^{n}f_{xi}^{2}}\sqrt{\sum_{i=1}^{n}f_{yi}^{2}}}. \end{array}$$

Following the computation of concept similarity, a series of candidate concepts *C*^+^ having a minimal cosine similarity {Cosine_sim(*C*_*xy*_*i*_^+^_), Cosine_sim(*C*_*xy*_*i*_^−^_)} is obtained. Using machine learning and the Hourglass of Emotions model developed by Plutchik in his research on human emotions, the framework categorizes emotions into different categories [42, 51] as sentic labels are constructed to denote each concept in VSM, and sentic API is used to forecast the comparable sentic levels for the eight emotion dimensions (positive and negative), suggested by the Cambria et al. [70]. If a match occurs, then the value of the particular emotional dimension is incremented. This procedure is repeated for all terms retrieved from the phrases in the review text to compute the emotions score using the following equations:

(3) $$\begin{array}{c} \text{Positiveemotions were measured as:}\\ \text{Joy}=\left(\frac{\text{Number ofwords associated with joy}}{\text{Number of conceptsembedded in a review}}\right)\times 100\text{,}\\ \text{Surprise}=\left(\frac{\text{Number ofwords associated with surprise}}{\text{Number of conceptsembedded in a review}}\right)100\text{,}\\ \text{Anticipation}=\left(\frac{\text{Number ofwords associated with anticipation}}{\text{Number of conceptsembedded in a review}}\right)\times 100\text{,}\\ \text{Trust}=\left(\frac{\text{Number ofwords associated with trust}}{\text{Number of conceptsembedded in a review}}\right)\times 100\text{,}\\ \text{Negative emotions were measured as:}\\ \text{Angry}=\left(\frac{\text{Number ofwords associated with angry}}{\text{Number of conceptsembedded in a review}}\right)\times 100\text{,}\\ \text{Anxiety}=\left(\frac{\text{Number ofwords associated with anxiety}}{\text{Number of conceptsembedded in a review}}\right)\times 100\text{,}\\ \text{Sadness}=\left(\frac{\text{Number ofwords associated with sadness}}{\text{Number of conceptsembedded in a review}}\right)\times 100\text{,}\\ \text{Disgust}=\left(\frac{\text{Number ofwords associated with disgust}}{\text{Number of conceptsembedded in a review}}\right)\times 100\text{.} \end{array}$$

### 4.4. Empirical Analyses

According to the descriptive statistics listed in Table 2, the average quality rating is 4.59, reflecting the maximum users who expressed positive sentiments about the service quality of healthcare provider. The average readability score of reviews is 9.74 based on 69.80 average length for these reviews. In addition, each doctor has an average of 308.11 reviews. Also, an average opinion score of a review is 0.79, an average RH score is 0.84, and an average review life is found to be 1682 days. Our data set contains 89 percent of medical doctors. A significant number of doctors are recent graduates of the top 100 medical schools in the U.S.

An important decision at this point was the regression model to use, given the dependent variable's limited low and high extremes. In line with previous research [38, 47], we used the TOBIT regression model because the sample and dependent variable were both censored (*Helpfulness*) [71, 72], based on the ratio of helpful votes to the overall vote count (ranges from 0 to 1) [38]. As a result, the dependent variable RH meets minimum dependent variables (censored data) requirements. This means the dependent variable does better than the censored value, which means the standard model of linear regression can be used [71].

The data were analyzed with STATA software, and the likelihood ratio and Efron's pseudo-R-square value were used to determine the goodness of fit [73]. Furthermore, the empirical analyses must be adjusted using the log-transformation in order to improve the fit of the variables in the empirical model and to adjust for overdispersion. We applied logarithmic transformation [74] to variables such as *helpfulness*, *wordiness*, *quality*, *popularity*, and *age*. To avoid having logarithms of zeros, the value of 1 is added to these variables [75]. All variables utilized to predict the RH are listed in equation (4). The description and measurement of variables are provided in Sections 4.2 and 4.3 and Table 1.

(4) $$\begin{array}{c} \ln\left({\text{Helpfulness}}_{i}\right)=\beta_{o}+\beta_{1}{\text{Readibility}}_{i}+\beta_{2}\ln\;.{\text{Wordiness}}_{i}+\beta_{3}{\text{Emotions}}_{i}+\beta_{4}\ln\;.{\text{Quality}}_{i}+\beta_{5}\ln\;.{\text{Popularity}}_{i}+\\ \beta_{6}\left({\text{Readibility}}_{i}{\times \text{DiseaseType}}_{i}\right)+\beta_{7}\left({\text{Wordiness}}_{i}{\times \text{DiseaseType}}_{i}\right)+\beta_{8}\left({\text{Emotions}}_{i}{\times \text{DiseaseType}}_{i}\right)+\\ \beta_{9}\left(\ln\;.{\text{Quality}}_{i}{\times \text{DiseaseType}}_{i}\right)+\beta_{10}\left(\ln\;.{\text{Popularity}}_{i}{\times \text{DiseaseType}}_{i}\right)+\beta_{11}{\text{Controls}}_{i}+\mu_{i}. \end{array}$$

### 4.5. Classification Techniques and Evaluation Metrics

The data mining software Weka 3.8.5 was employed in this study, and the classification model was constructed using a support vector machine (SVM), linear regression (LR), random forest (RF), and gradient boost decision tree (GBDT). We chose these models because previous studies used these models successfully and achieved excellent classification results [18, 31].

SVM is based on statistical learning theory and is now one of the most successful approaches for analyzing high-dimensional datasets and is extensively used to perform classification tasks [76]. The fundamental notion of SVM is the application of structural risk minimization, which reduces boundary error through induction while minimizing overall risk. Once the data are transferred to a higher-dimensional space, they are separated by a hyperplane. A hyperplane-projected subspace can map a new instance, which can then be allocated to the majority class in that subspace.

Regression analysis refers to the statistical technique used to analyze data. Its objective is to ascertain the degree of correlation between two or more variables and build a mathematical model for forecasting the outcome. LR is a nonlinear regression model that attempts to predict how likely an event will happen by fitting data to a logistic function. This allows inputs with any value to be converted and confined to a value between 0 and 1.

A RF is a technique for ensemble learning that was established through the construction of numerous DTs [77]. Training an RF involves bagging bootstrap cases and then selecting a random subset of features. Following that, a set of DTs is generated using each bootstrap instance set containing a subset of features. After the set of trees has been built, the majority class of individual trees can be used to make a prediction about samples that have yet to be seen.

Gradient boost decision tree algorithm systematically adds weak learners in such a way that each new learner matches the preceding step's residuals, hence improving the model [78]. The final model combines the outcomes of each phase to produce a strong learner. Gradient boosted decision trees technique makes use of decision trees as week learners to achieve better results. The residuals are detected using a loss function. It is worth mentioning that when a new tree is added to the model, the current trees remain unchanged. The residuals from the existing model are well-fit by the decision tree that has been introduced. The effectiveness of applied learning classifiers is assessed using two assessment measures (f-measure and accuracy). These metrics are mathematically defined as follows:

(5) $$\begin{array}{c} \text{Accuracy}=\frac{\text{TP}+\text{TN}}{\text{TP}+\text{TN}+\text{FP}+\text{FN}},\\ \text{Precision}=\frac{\text{TP}}{\text{TP}+\text{FP}},\\ \text{Recall}=\frac{\text{TP}}{\text{TP}+\text{FN}},\\ F1=2\times \frac{\text{precision}\times \text{recall}}{\text{precision}+\text{recall}}. \end{array}$$

## 5. Results

Prior to conducting a tobit analysis, this research conducted several diagnostic tests to determine the model's heteroscedasticity and multicollinearity. The maximum variance inflation factors (VIF) range between 1.53 and 5.51, much below the cut-off value of 10, indicating that multicollinearity is not an issue at the moment [79]. Furthermore, the correlation values between variables suggest that our dataset is free of multicollinearity (0.90 and higher) [50]. In addition, we calculated standard errors for our models that were consistent with heteroscedasticity (see Table 3). With a relatively substantial likelihood ratio, our model attained goodness of fit (*p* ≤ 0.001) and McKelvey and Zavoina [73] Efron's Pseudo *R*^2^ value of 0.083.

In particular, when looking at the major effects for review-related signals in Table 3, the findings of the regression analysis suggest that the *readability* coefficient is positively significant (*β* = 0.174, *p* < 0.05). As a result, *H1* is supported, which is in line with earlier research findings [19]. Following that, the results indicate a substantial positive coefficient for *wordiness* (*β* = 0.320, *p* < 0.05), which supports *H2*, consistent with the findings of Mudambi and Schuff [38], but negates the results of Qazi et al. [15]. Furthermore, the significant positive coefficient for *emotions* (*β* = 0.013, *p* < 0.001) demonstrates that the association will be stronger when both positive (including joy, sadness, trust, except surprise) and negative emotions (including anger, disgust, fear, except anticipation) are included in a review, accepting *H3*. These findings are in line with earlier research [51]. We find support for *H4* when we looked at the service-related signals (*β* = 0.125, *p* < 0.05), emphasizing that when a POR focuses on the quality of service *quality*, its helpfulness value increases. These findings corroborate prior findings [14]. Moreover, we uncover evidence supporting *H5*, which hypothesizes a considerable association between service popularity and RH. In particular, the statistically significant positive coefficient of service *popularity* (*β* = 0.232, *p* < 0.01) demonstrates that there is a positive association between the number of reviews and helpful votes obtained by the doctor. These findings are consistent with those of Zhang and Lin [40].

When it comes to the moderating effect of illness severity, the results show that they strongly support the use of interaction terms (*Wordiness* × *Disease Type*), (*Quality* × *Disease Type*) and (*Popularity* × *Disease Type*) as *H7*, *H9*, and *H10*, indicating that having more words in a review, as well as its quality and popularity of a physician service embedded in the review, has a more significant positive impact on how people think about the RH for serious diseases than for mild diseases. However, our findings do not provide support for H6 and H8. There is no evidence found to support the hypothesis that disease type moderates the influence of readability or emotion on the perceived RH. Readability and emotion both contribute equally to the perceived RH for different disease conditions. Furthermore, the results for the control variables are consistent with earlier findings [28]. The coefficient (*β*) for age, title, education, and experience is significant for both disease conditions.

To maximize the practical value of our research, we used a text mining strategy to estimate the efficacy and performance of our suggested multimethod model by taking into account all of the signals from a review and service at the same time. For the purpose of testing different classification models, the data mining software Weka 3.8.5 was employed. Based on the number of helpful votes received by a review, we classify our reviews as “helpful” and “not helpful” groups and employ strategies to develop the classification model detailed in Table 4. In particular, a review is considered helpful if it receives at least one vote. The model estimation is performed using the training data and the model is validated using the holdout sample to see if the model is useful for physicians and to predict physician RH. This practice contributes to the avoidance of overfitting.

We classified PORs as helpful or not helpful based on review- and service-related attributes using well-known machine learning methods. The predictive models are built and 10-fold cross-validation is used to compare the accuracy of the predictive models across all experiments. The hybrid set of review- and service-related features (combination of readability, wordiness, emotions, quality, and popularity) is used to train four different learning algorithms. The results of experiments using a hybrid set of features are summarized in Table 4.

Using a hybrid set of features to predict physician RH, PORs dataset delivers 73.10% accuracy and 73.14% f-measure with SVM classifier. Next, using a hybrid set of review- and service-related features, the PORs dataset outputs 75.22% accuracy and 75.11% f-measure with the LR classifier, 81.13% accuracy and 81.15% f-measure with the RF, and 91.12% accuracy and 91.63% with GBDT classifier. The experimental results demonstrate that the overall performance of the model is rather promising, demonstrating the applicability of the suggested hybrid features in terms of accuracy and f-measure metrics for RH prediction. The proposed hybrid features model developed by this study clearly outperforms the other models, successfully classifying 91.12% of all cases correctly. A series of experiments are carried out with the machine learning classifier because it demonstrated the best performance compared to the models analyzed in the previous studies [16, 31, 40]. All of the evaluation parameters indicate that our suggested model performs well in terms of predicting physician RH.

## 6. Discussion

PRWs offer patients a place to talk about their healthcare experiences or write reviews online [80]. On social media networks, patients post thousands of reviews and remarks about their experiences, making it difficult to discern which reviews are useful and which are not. Therefore, it is vital to investigate characteristics and establish a study technique for implicitly classifying helpful reviews rapidly and reliably. For this study, we collected a vast number of PORs from Healthgrades.com. A further investigation was carried out into the impact of three review- and two service-related signals on perceived RH. Both the review- and service-related signals on PRWs significantly influence perceived RH, in line with previous studies [10, 13, 23]. Inspired by earlier studies [25, 28], we examined the moderating influence of disease type in the connection between review- or service-related signals and RH. The impact of wordiness, quality, and popularity on the perception of RH is moderated by the disease type. Moreover, we used data mining approaches to construct multiple classification models for the assessment of RH. The results indicate that our proposed model performs exceptionally well at classifying and predicting physician RH.

The findings show that our proposed model for review- and service-related signals successfully influences perceived RH, which has various theoretical implications. From a theoretical point of view and to the best of our knowledge, this research is the first to explore the effects of review- and service-related signals on online physician RH.

By and large, these findings advance the state of the art in estimating helpfulness, which has historically concentrated on the local features of reviews [38, 47, 81]. The unique thing about our work is that we look at patient feedback in a wider user or service-related context, taking into account a broader set of determinants. Moreover, this study advances signaling theory by demonstrating that the signaling environment (disease type) has an effect on review- and service-related signals. The findings fill a gap in the existing knowledge by identifying the characteristics of the helpful POR across a wide range of disease conditions. The results revealed a significant moderating relationship between wordiness, quality, popularity, and perceived RH. The dichotomy between a review- or service-related signals and perceived RH is predicted to be interpreted by disease type. Our proposed model, which is predictive in nature, enables online healthcare providers to prioritize the most helpful reviews. Our research findings confirm the practical significance of the suggested approach by indicating the classification performance while predicting RH. Researchers also claim that this is the first attempt to use a multimethod approach including implicit and domain knowledge-based specialized emotion analysis methodologies to predict physician RH. This study demonstrates the critical relevance of RH as a future source of health informatics research.

The findings of this research have a number of implications in practice. For the designers of Internet health rating websites, our approach includes a recommendation mechanism that suggests useful reviews for patients on PRW. It is possible to automatically identify the wants and requirements of patients while they explore the PRWs by searching for high to low levels of physician expertise, top-rated physicians, verified physicians, and selecting doctors based on their star ratings. We anticipate that this intelligent recommendation system will result in more helpful physician reviews based on the readability, depth, specific emotions, service quality, and popularity-related attributes of the service. Recommendation systems like this would save patients time and money when they use PRWs to find helpful reviews for providers they were about to visit. For healthcare providers, administrators and patients are able to view the useful assessments of their healthcare services on health rating websites and online forums. Individuals are eager to search useful/helpful reviews that might assist them in determining the worth of healthcare services or choosing the best physician they want to visit. Furthermore, when reading a vast collection of reviews, patients and their caregivers frequently experience substantial cognitive processing costs. The contribution of this study provides the opportunity for healthcare practitioners and administrators to reduce the cognitive processing costs associated with PORs in order to improve their organization.

This study has certain limitations. First, it is quite challenging to choose an acceptable dataset for analyzing the determinants affecting online physician RH, given only a small percentage of reviews receive a helpful vote from reviewers. Although it is well-known and ranks among the highest-trafficked platforms in the U.S., other platforms that allow users to post reviews should also be taken into account. Second, future research could incorporate reviewers' behavior and its effect on predicting physician RH. Finally, future studies into physician RH should use more advanced and efficient text mining algorithms, such as deep learning (Neural Networks).

## Figures and Tables

**Table 1:** Variable description.

Variable	Variable name	Description	Measurement
Dependent variable	Review helpfulness	Review helpfulness refers to ratio of the number of helpful votes a review received to the total votes evaluating helpfulness of that review	Helpfulness
Independent variables	Review readability	Readability is the amount of efforts and educational level required to understand an online review, which is measured by the (1) automated readability index (ARI), (2) Coleman-Liau index (CLI), (3) Flesch-Kincaid grade level (FKGL), (4) Flesch-Kincaid reading ease (FKRE), (5) Gunning's Fog index (GFI), and (6) simple measure of gobbledygook (SMOG) readability index of review text	Readability
	Review wordiness	Review wordiness is the total number of concepts in a review, measured through concept extraction process of the sentic computing framework	Wordiness
	Review emotions	Review emotion is the average polarity of a review, which is measured by the average percentage of the number of positive and negative discrete emotions embedded in a review	Emotions
	Service quality	Service quality reflects the tone or preference of users expressed in positive, negative, or neutral opinion for the service, which is measured by the review valence as average number of rating-stars a service receives	Quality
	Service popularity	Service popularity reflects the number of users discussing the service, which is measured by the review volume as number of reviews received by a service	Popularity
Moderating variable	Disease type	The disease mortality rate in which a patient suffered from	Disease type
Control variables	Review age	For how long a review has been written on a PRW	Age
	Physician title	A professional title of a physician practicing in healthcare facility	Title
	Physician education	Rank of a medical school the physician graduated from	Education
	Physician graduation	Number of years since a physician graduated	Graduation
	Physician experience	Number of years since a physician is in practice	Experience

**Table 2:** Variables descriptive statistics.

Variables	Variable name	Min	Max	Mean	Std. Dev
Dependent variable	Review helpfulness	0.5	1.0	0.84	0.22
Independent variables	Review readability	1	12	9.74	1.02
	Review wordiness	8	94	69.80	2.11
	Review emotions	0	1.0	0.79	0.24
	Service quality	1	5	4.59	1.26
	Service popularity	3	412	308.11	3.41
Moderating variable	Disease type	0	1	0.462	0.19
Control variables	Review age	0	1826	1682	91.23
	Physician title	0	1	0.89	0.23
	Physician education	0	1	0.86	0.29
	Physician graduation	0	26	4.70	0.62
	Physician experience	0	25	4.10	0.54

**Table 3:** Heteroscedasticity compatible results of hypotheses testing.

Constructs	Β	Std. error	*p* value	*t* value
(Constant)	1.993	0.071	0.006^∗∗^	30.401
Age	0.010	0.015	0.016^∗^	0.041
Title	0.036	0.025	0.002^∗∗^	0.087
Education	0.465	0.970	0.000^∗∗∗^	1.432
Graduation	0.150	0.533	0.312	1.196
Experience	0.028	0.078	0.007^∗∗^	0.125
Readability	0.174	0.006	0.043^∗^	3.423
Wordiness	0.320	0.041	0.030^∗^	2.732
Emotions	0.013	0.001	0.000^∗∗∗^	1.230
Quality	0.125	0.015	0.045^∗^	1.014
Popularity	0.232	0.033	0.004^∗∗^	2.006
Disease type	0.024	0.041	0.012^∗∗^	0.013
Readability × disease type	0.028	0.010	0.366	0.750
Wordiness × disease type	0.018	0.009	0.038^∗^	0.900
Emotion × disease type	0.040	0.004	0.256	0.430
Quality × disease type	0.080	0.019	0.036^∗^	0.548
Popularity × disease type	0.053	0.010	0.030^∗^	0.430
Efron's *R*^2^	0.084	Log-likelihood	−2745.618	
Likelihood ratio	429.631		*p* ≤ 0.001, df = 8	

Note: ^*∗*^*p* < 0.05, ^*∗∗*^*p* < 0.01, ^*∗∗∗*^*p* < 0.001.

**Table 4:** Measuring classification performance and comparing models.

Algorithms	Accuracy	Precision	Recall	*F*-measure	Previous models	Accuracy	Precision
Support vector machine	73.10	73.05	73.25	73.14	Lee et al. [31]	84.30%	—
Linear regression	75.22	74.12	76.19	75.11	Eslami et al. [16]	69.0%	—
Random forest	81.13	80.35	82.03	81.15	Zhang and Lin [40]	—	85.19%
Gradient boost decision tree	91.12	91.07	92.18	91.63	Proposed model	91.12%	—

## Data Availability

The data used to support the findings of this study are available from the corresponding author upon request.
